# Coupled Multi-Physics Study on SF_6_ Decomposition Gas Diffusion and Sensor Placement Optimization in GIS Busbars

**DOI:** 10.3390/s26185782

**Published:** 2026-09-11

**Authors:** Duohu Gong, Niyar Di, Yadi Xie, Shan Li, Ruyue Mai, Tong Li, Qian Shi

**Affiliations:** Electric Power Research Institute of State Grid Xinjiang Electric Power Co., Ltd., Urumqi 830011, China

**Keywords:** GIS fault diagnosis, SF_6_ decomposition products, multi-physics coupled simulation, sensor placement optimization, diffusion characteristics

## Abstract

**Highlights:**

**What are the main findings?**

- A model for SF_6_ byproduct diffusion in GIS busbars (fault location, species, initial concentration) was validated for simulating rise, peak, and decay.
- Diffusion is governed by fault location and gas properties; sensor optimization uses response time and peak concentration, with point M4 best overall.

**What are the implications of the main findings?**

- The findings provide a theoretical basis and simulation reference for analyzing the diffusion behavior of SF6 decomposition products in GIS gas chambers, selecting fault gas monitoring points, and identifying early-stage defects.
- The optimized placement results indicate that M4 can be selected as the preferred monitoring point when only one sensor is monitoring scenarios, and M2 can serve as an auxiliary monitoring point to improve early fault identification capability.

**Abstract:**

Traditional fault diagnosis methods for gas-insulated switchgear (GIS) equipment primarily rely on offline detection and periodic maintenance, which suffer from limitations such as poor real-time performance and localization difficulties, thereby compromising the safe and stable operation of ultra-high-voltage power grids. To enhance the accurate identification and localization capabilities of defects within GIS equipment, this study first establishes a multi-physics coupled simulation model integrating temperature field, flow field, and concentration field to analyze gas diffusion characteristics under varying conditions of fault source locations, decomposition product types, and initial concentrations. Subsequently, a GIS busbar gas chamber experimental platform is constructed to validate the simulation model. Finally, a response time matrix, a peak concentration matrix, and a fault coverage index are developed, and a weighted comprehensive evaluation method is employed to optimize sensor placement schemes. The findings reveal that fault source location significantly influences concentration response speed and spatial distribution patterns; SO_2_, HF, H_2_S, and SOF_2_ exhibit distinct diffusion characteristics due to their differing physical properties; and initial concentration primarily affects the non-uniformity during the early diffusion stage. The simulation results demonstrate good agreement with experimental data, with a maximum root-mean-square error of 3.936 × 10^−4^. Monitoring point M4 achieves the highest comprehensive score, making it the preferred location for single-sensor deployment. These results provide a theoretical foundation and technical guidance for GIS online monitoring and fault diagnosis.

## 1. Introduction

Gas-insulated switchgear (GIS) has been extensively deployed in high-voltage and ultra-high-voltage power systems due to its compact structure, high operational reliability, and minimal maintenance requirements [1]. However, its fully enclosed configuration makes internal defects difficult to detect directly, thereby posing threats to the secure and stable operation of power grids. SF_6_ gas, serving as the primary insulation and arc-quenching medium in GIS equipment, undergoes decomposition under defect conditions such as partial discharge [2], overheating, or arcing. These decomposition processes, coupled with reactions involving trace amounts of moisture, oxygen, and metallic materials, generate decomposition products including SO_2_, SOF_2_, and CF_4_ [3]. The types of and concentration variations in these decomposition products are closely correlated with the internal insulation condition of the equipment. Consequently, online monitoring based on SF_6_ decomposition products has emerged as a crucial approach for GIS condition assessment and fault diagnosis [4,5,6].

Online monitoring of SF_6_ decomposition products relies on sensors capturing concentration variations in characteristic gases. However, the concentration signals acquired by sensors do not directly represent the gas generation information at the fault source; rather, they reflect localized responses formed after decomposition products undergo diffusion, convection, and mixing within the gas chamber. These responses are collectively influenced by factors including fault source location, gas chamber geometry, temperature field distribution, and flow field patterns, resulting in significant variations in fault discrimination capability across different monitoring positions [7,8,9]. Therefore, it is essential to optimize sensor placement within GIS busbar gas chambers based on a comprehensive understanding of SF_6_ decomposition product migration and diffusion mechanisms.

Considerable research has been conducted on the migration and diffusion characteristics of SF_6_ decomposition products within GIS busbar gas chambers. Shen Leyu, Liu Ming, and colleagues [10,11] employed three-dimensional mass transfer models to describe the diffusion processes of components such as SOF_2_, SO_2_F_2_, and CF_4_ under varying gas generation rates and defect locations, revealing the gradual attenuation pattern of decomposition product concentrations along the propagation direction [12]. He Yi et al. [13] simulated the diffusion behavior of SO_2_ within GIS using computational fluid dynamics methods, demonstrating that diffusion processes alter the characteristic component content at detection points, thereby affecting fault diagnosis accuracy. Ren Zetong et al. [14] obtained distribution characteristics of CO_2_, CF_4_, and C_2_F_6_ as functions of discharge duration and defect location through experimental and simulation comparisons, validating the applicability of three-dimensional mass transfer models. Nevertheless, these studies predominantly operate under isothermal assumptions, considering only molecular diffusion driven by concentration gradients while inadequately accounting for natural convection effects induced by conductor heating during operation. In practical operation, GIS busbars carrying load currents generate temperature rises due to conductor resistance losses, establishing temperature gradients between conductors and enclosures that induce natural convection within the gas chamber [15]. When equipment experiences abnormalities such as poor contact or localized overheating, localized heat sources further modify the flow field structure, transforming the decomposition product transport process from pure molecular diffusion into a complex process involving both molecular diffusion and convective transport [16]. Existing research indicates that coupling effects among temperature field, flow field, and concentration field alter the spatial distribution patterns of decomposition products, causing pronounced variations in response times and peak concentrations at different monitoring points [17,18]. Therefore, neglecting natural convection and temperature rise effects may result in diffusion predictions that fail to accurately reflect the actual migration characteristics of decomposition products within GIS gas chambers, consequently compromising the reliability of sensor placement evaluations.

The core objective of sensor placement optimization is to enable a limited number of monitoring points to cover primary fault scenarios as comprehensively as possible while obtaining rapid, effective, and discriminative responses following fault occurrence. Jiang Dongnian et al. [19] achieved optimal sensor configuration through diagnosability evaluation methods based on Kullback–Leibler divergence. Zhang et al. [20] proposed dual coverage and reliability indices incorporating redundant voting mechanisms, enabling the system to maintain high detection performance even after single-sensor failure. An Weipeng et al. [21] utilized multi-objective evolutionary algorithms such as NSGA-II to balance relationships among sensor quantity, detection performance, and deployment cost. However, these methods are predominantly applied in chemical process or structural monitoring contexts, lacking application cases for online monitoring of SF_6_ decomposition products in GIS gas chambers. Moreover, the foundational optimization data typically derive from simplified assumptions and lack support from multi-physics coupled diffusion simulations that incorporate actual operating conditions.

Accordingly, this study focuses on GIS busbar gas chambers to investigate SF_6_ decomposition product diffusion characteristics and sensor placement optimization under multi-physics coupled conditions. First, a multi-physics coupled simulation model integrating temperature field, flow field, and concentration field is established to analyze the diffusion patterns of SO_2_, HF, H_2_S [22], and SOF_2_ within busbar gas chambers under varying fault source locations, decomposition product types, and initial concentration conditions, thereby obtaining concentration variation characteristics at each simulated monitoring position. Subsequently, a GIS busbar gas chamber experimental platform is constructed to validate the accuracy of the diffusion simulation model. Finally, comprehensive evaluation indices are formulated based on response time, peak concentration, and fault coverage, employing weighted comprehensive evaluation methods to optimize sensor placement schemes. The research findings provide theoretical foundations and engineering references for the design of online monitoring systems and fault diagnosis in GIS equipment.

## 2. Multi-Physics Coupled Model Development

### 2.1. Geometric Model and Fundamental Assumptions

**Figure 1:**
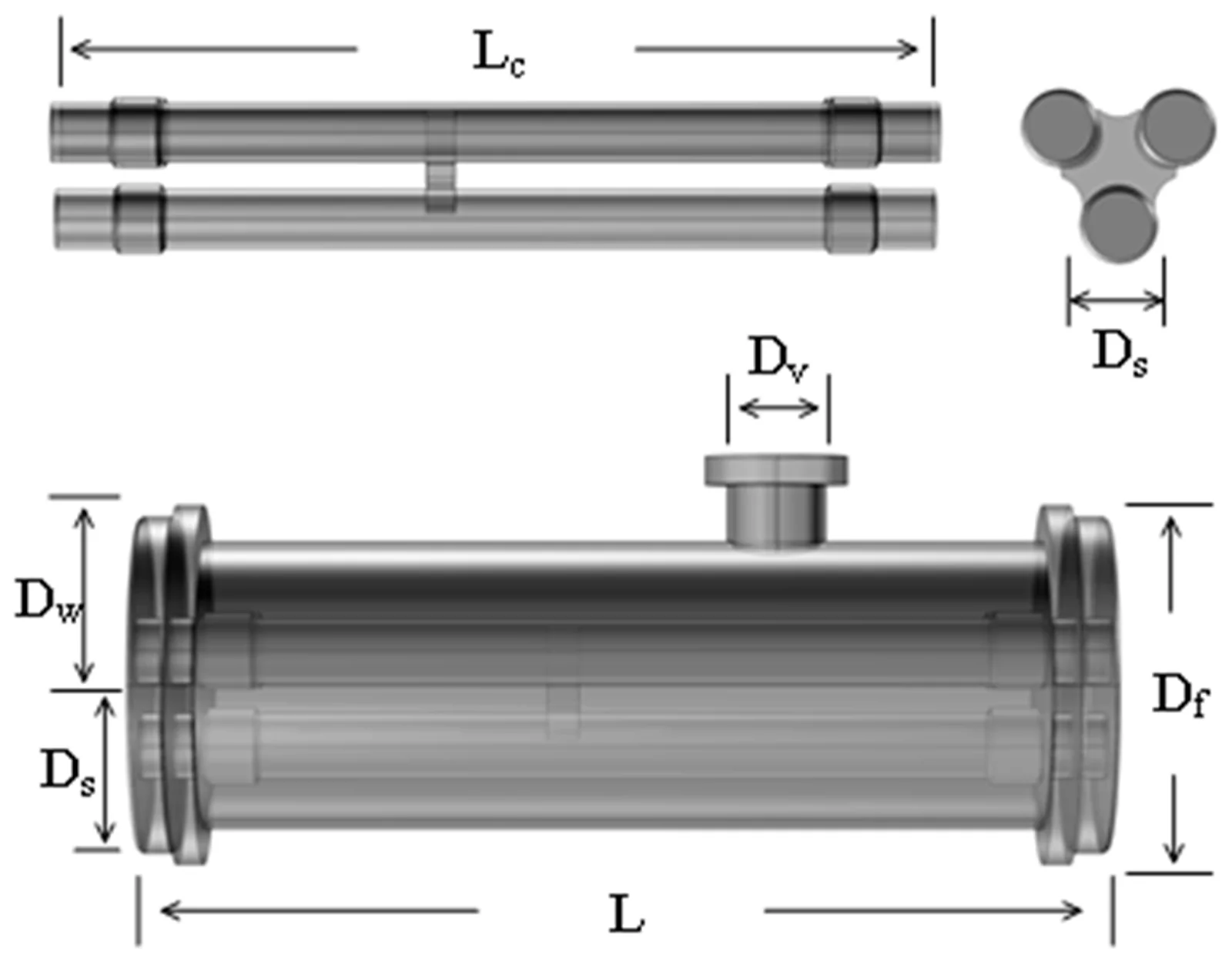
Geometric model and computational domain of the busbar air chamber.

Figure 1 presents the three-dimensional computational model for SF_6_ decomposition gas diffusion response within the GIS busbar gas chamber. The model comprises four components: a metallic enclosure, a central conductor, a post insulator, and a gas computational domain. Since this study focuses on analyzing the convection–diffusion processes of SF_6_ decomposition gases within the busbar gas chamber under typical micro-defect conditions and the response characteristics of candidate monitoring points, local structural features such as bolts, chamfers, mounting holes, and flange connections are omitted during the modeling process to reduce geometric complexity. These simplifications have minimal impact on the primary flow field and concentration field distributions. The principal structural parameters of the GIS busbar gas chamber simulation model are listed in Table 1. SF_6_ gas fills the entire enclosed gas chamber, and decomposition gas release sources are positioned in regions near the central conductor, near the post insulator, and beneath the main sampling port according to typical micro-defect locations [23]. To emphasize the diffusion patterns of decomposition gases and monitoring point response characteristics, the following assumptions are established: SF_6_ gas is treated as a continuous medium, and gas flow is considered low-speed flow; decomposition gas concentrations are sufficiently low that they do not affect the existing temperature field and natural convection flow field within the gas chamber; the enclosure, central conductor, and insulator walls all adopt no-slip boundary conditions; and decomposition gases satisfy zero-flux conditions at wall surfaces.

### 2.2. Governing Equations for Temperature Field, Flow Field, and Concentration Field

Using the conductor, enclosure, and gas chamber as the computational domain, a three-dimensional non-isothermal laminar flow model is established to accurately capture thermal–flow coupling characteristics. The temperature field satisfies the energy conservation equation [24]:

(1) $$\rho C_{p}\left(\frac{\partial T}{\partial t}+u\cdot \nabla T\right)=\nabla \cdot \left(k\nabla T\right)+Q_{\text{source}}$$

where ρ is the gas density, in kg·m^−3^; C_p_ is the specific heat capacity at constant pressure, in J·kg^−1^·K^−1^; k is the thermal conductivity, in W·m^−1^·K^−1^; u is the fluid velocity, in m·s^−1^; and Q_source is the heat source term. The boundary conditions for the temperature field are specified as follows: the conductor surface is treated as a constant heat-flux boundary, the inner wall of the enclosure is defined as a convection–radiation coupled boundary, and the initial temperature T_0_ is set to 25 °C. The specific gas property parameters used in the model are listed in Table 2 [25,26].

The flow field is described using a laminar flow model under the Boussinesq approximation, and is governed by:

(2) ∇⋅u=0

(3) $$\rho \left(\frac{\partial u}{\partial t}+u\cdot \nabla u\right)=-\nabla p+\mu \nabla^{2}u+\rho_{0}\beta \left(T-T_{0}\right)g$$

where μ is the dynamic viscosity, in Pa·s; β is the thermal expansion coefficient, $\beta =-\frac{1}{\rho }{\left(\frac{\partial \rho }{\partial T}\right)}_{p}$; and g is the gravitational acceleration, in m·s^−2^. The wall satisfies the no-slip boundary condition, u = 0.

To characterize the diffusion behavior of the SF6 decomposition gases SO_2_, SOF_2_, H_2_S, and HF generated by micro-defects within the gas chamber, a transport of diluted species model [27] is introduced. A source term is specified at the release location to represent the continuous generation of decomposition gases. The gas diffusion coefficients are listed in Table 3.

For the nth decomposition gas, its mass concentration C_n_ satisfies:

(4) $$\frac{\partial c_{n}}{\partial t}+\nabla \cdot \left(uc_{n}\right)=\nabla \cdot \left(D_{n}\nabla c_{n}\right)+R_{n}\left(x,t\right)$$

where

(5) $$R_{n}\left(x,t\right)=\left\{\begin{array}{c} a_{k}\cdot b_{i,n}\cdot S_{0}\cdot \delta \left(x-x_{i}\right),t>t_{0}\\ 0,\;t<t_{0} \end{array}\right.$$

### 2.3. Coupled Solution and Grid Independence Verification

#### 2.3.1. Coupled Solution Strategy

To reduce the computational complexity of transient multiphysics simulations, a weakly coupled solution strategy is adopted in this study. First, the steady-state thermal–flow field is solved in the absence of decomposition gases to obtain the background SF6 natural convection driven jointly by conductor heating and shell heat dissipation. The resulting steady flow field is then used as the velocity input for the mass transfer calculation to solve the transient convection–diffusion process of low-concentration species such as SO_2_, SOF_2_, H_2_S, and HF after release from micro-defects. Because the concentration of decomposition gases is low, their generation and diffusion do not significantly alter the temperature field or flow field structure inside the gas chamber; therefore, the feedback effect of the concentration field on the thermal–flow field can be neglected. The influence of temperature on the mass transfer process is mainly reflected through the natural convection velocity field and the temperature-dependent diffusion coefficient D_n_(T).

#### 2.3.2. Grid Independence Verification

To balance computational accuracy and efficiency, grid independence verification was conducted for the established multiphysics model of the GIS busbar gas chamber. The computational domain was discretized using an unstructured tetrahedral mesh, with local refinement applied in regions with large temperature and velocity gradients, including the conductor surface, the inner wall of the enclosure, and the vicinity of the basin insulator. In addition, boundary-layer meshes were generated at the solid–fluid interfaces to improve the accuracy of gradient calculations in the near-wall region.

Under identical boundary conditions and solver settings, four computational models with different mesh densities were established, and the maximum conductor temperature T_max_ and the maximum gas velocity u_max_ in the chamber were selected as the evaluation indices for grid independence. Here, T_max_ is used to characterize the stability of the calculated results for conductor heating and solid–gas coupled heat transfer, while u_max_ reflects the sensitivity of the SF_6_ natural convection field, driven by temperature gradients, to the mesh scale. The calculated values of the key parameters under different mesh schemes are listed in Table 4.

## 3. Analysis of the Diffusion Characteristics of SF_6_ Decomposition Gases

### 3.1. Diffusion Characteristics Under Different Fault Source Locations

**Figure 2:**
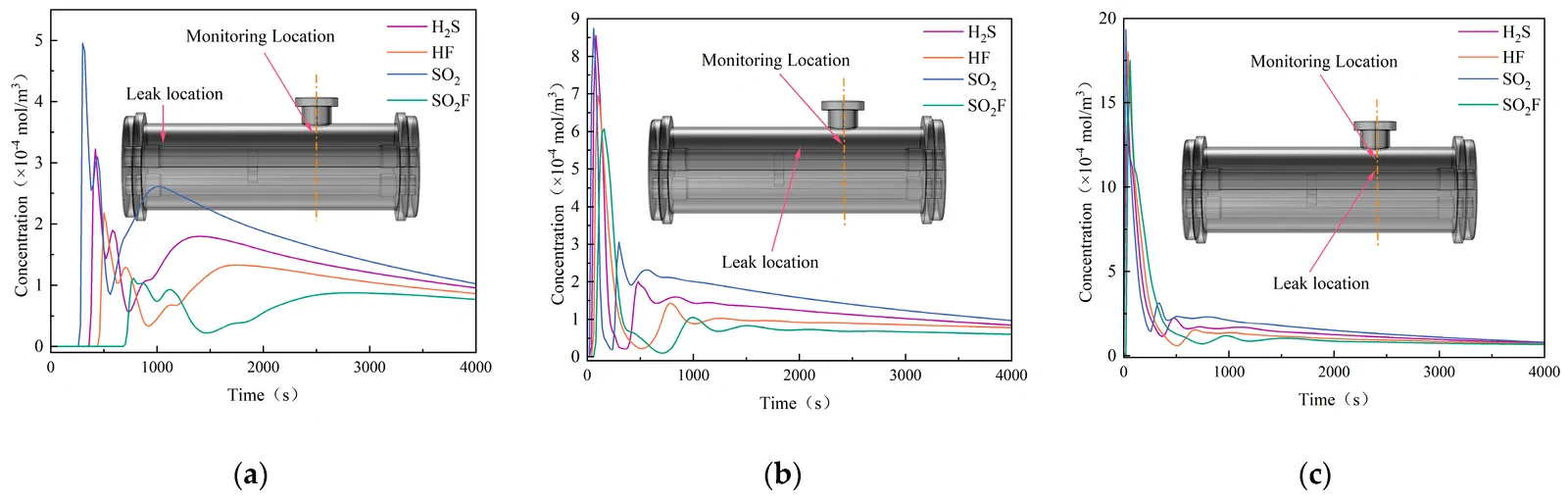
Time-dependent diffusion concentration profiles of HF, H_2_S, SO_2_, and SOF_2_: (**a**) at the basin insulator; (**b**) in the middle of the chamber; (**c**) below the main sampling port.

The temporal evolution of the concentrations of HF, H_2_S, SO_2_, and SOF_2_ at the main sampling port for three fault locations, namely, near the basin insulator, in the middle of the chamber, and below the main sampling port, is shown in Figure 2. The release concentration of each decomposition product at the fault source is 0.08 mol/m^3^ in all cases (the same applies to Section 2.2). The results show that the fault source location has a significant influence on the concentration response at the monitoring point.

When the fault source is located near the basin insulator, the distance to the main sampling port is relatively large, and the decomposition gases experience more pronounced diffusive dilution during transport. As a result, the concentration response exhibits a certain delay and a relatively low peak value. When the fault source is located in the middle of the chamber, the rise rate and peak concentration of each component are at an intermediate level, and the concentration decays more gradually after the release stops. When the fault source is located below the main sampling port, the gases can reach the monitoring point within a short time, leading to the fastest response and the highest peak concentration. However, after the release ceases, the locally accumulated high-concentration gas diffuses rapidly, and the concentration decreases most noticeably.

Overall, the closer the fault source is to the main sampling port, the faster the monitored concentration response and the higher the peak value. Among the different components, SO_2_ generally shows the highest concentration, HF and H_2_S are relatively close to each other, and SOF_2_ remains comparatively lower.

Figure 4 shows the diffusion contours of H_2_S for different fault source locations. It can be seen that when the fault source is located near the basin insulator, H_2_S mainly accumulates at the end region and diffuses axially toward the interior of the gas chamber, forming a distinct concentration gradient. When the fault source is located in the middle of the chamber, H_2_S diffuses toward both sides and exhibits a relatively balanced distribution. When the fault source is located below the main sampling port, a high-concentration region quickly forms near the sampling port, resulting in the fastest local response.

**Figure 3:**
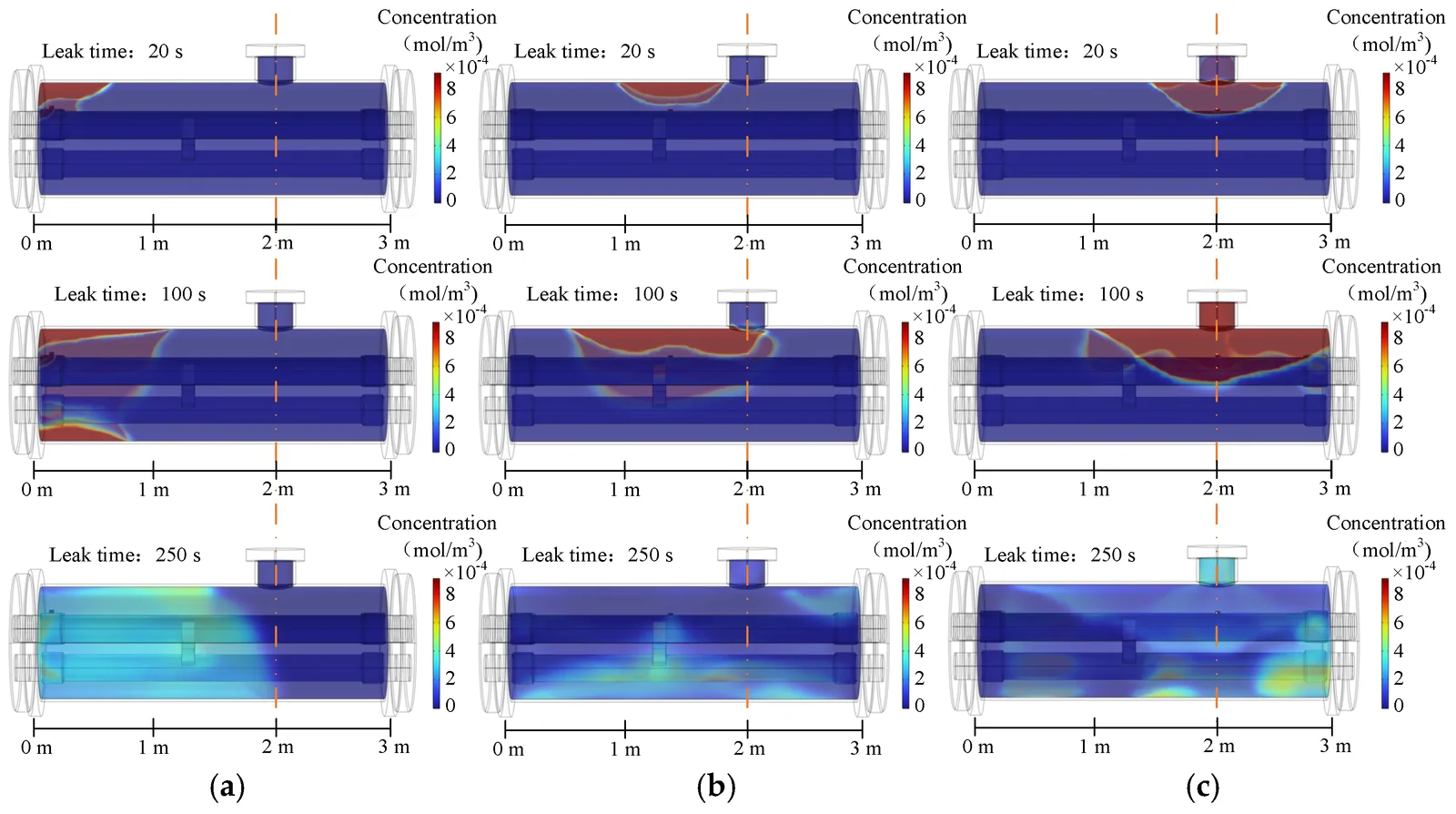
Diffusion cloud map of decomposition product H_2_S: (**a**) at the basin insulator; (**b**) in the middle of the chamber; (**c**) below the main sampling port.

Overall, the closer the fault source is to the sampling port, the faster the monitoring response and the higher the local concentration, but the more non-uniform the overall concentration distribution within the gas chamber. In contrast, faults located at the end region exhibit a more pronounced axial diffusion characteristic. Taken together, Figure 2 and Figure 3 indicate that the fault source location not only affects the spatial diffusion behavior of the decomposition products inside the gas chamber, but also significantly alters the concentration response at the main sampling port.

### 3.2. Diffusion Characteristics of Different Decomposition Products

**Figure 4:**
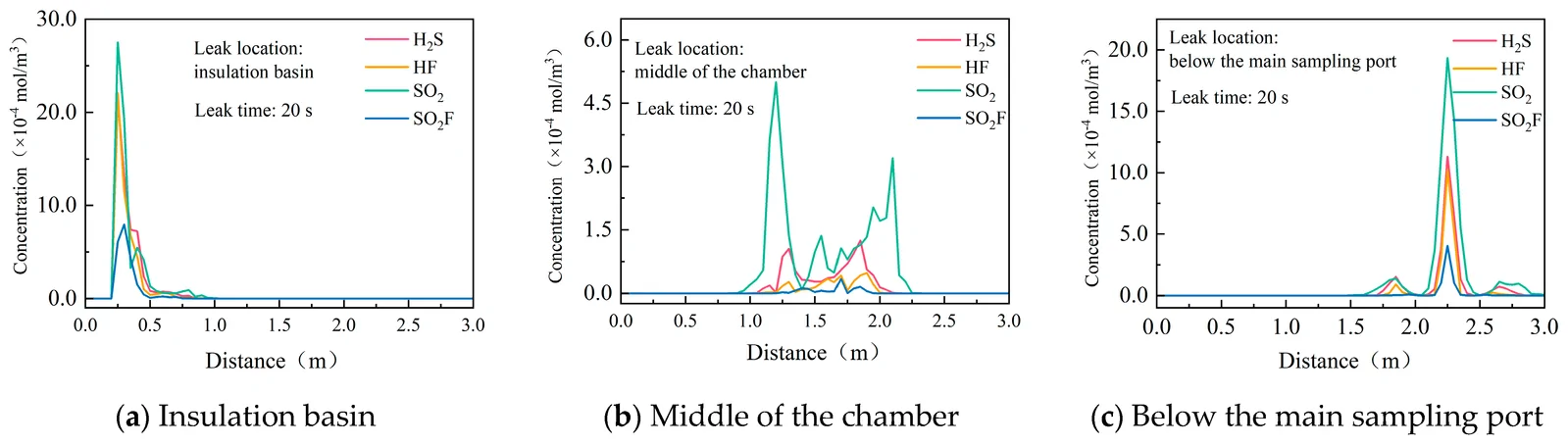
Spatial concentration distribution of HF, H_2_S, SO_2_, and SOF_2_ decomposition products at the three fault locations. (**a**) insulation basin, (**b**) middle of the chamber, and (**c**) below the main sampling port.

Figure 4 presents the axial concentration distribution curves of HF, H_2_S, SO_2_, and SOF_2_ within 20 s for the three fault locations. It can be seen that the concentration peaks of all decomposition products are concentrated near the fault source, and the peak positions shift with the change in fault source location. This indicates that, at the initial stage of release, the gases are not yet fully mixed and exhibit obvious local accumulation characteristics.

Among the different components, SO_2_ shows the highest local peak concentration, followed by HF and H_2_S, while SOF_2_ has the lowest peak value. This suggests that there are clear differences in the migration and dilution behaviors of these gases in SF_6_. Combined with Table 2 and Table 3, these differences are mainly related to physical properties such as diffusion coefficient, density, and viscosity. Owing to its relatively balanced physical property combination, SO_2_ is more likely to form a locally high-concentration region near the fault source. Although HF has a relatively large diffusion coefficient, it still exhibits a certain degree of local accumulation in the short term due to the influence of parameters such as viscosity. The diffusion coefficient of H_2_S is second only to that of HF, so its diffusion is relatively more sufficient, and its concentration peak is lower than those of SO_2_ and HF. SOF_2_, having the smallest diffusion coefficient, exhibits a comparatively weaker axial concentration response.

Overall, the axial concentration distribution of the decomposition products is jointly determined by the fault source location and the physical properties of the gases.

### 3.3. Diffusion Characteristics at Different Initial Concentrations

**Figure 5:**
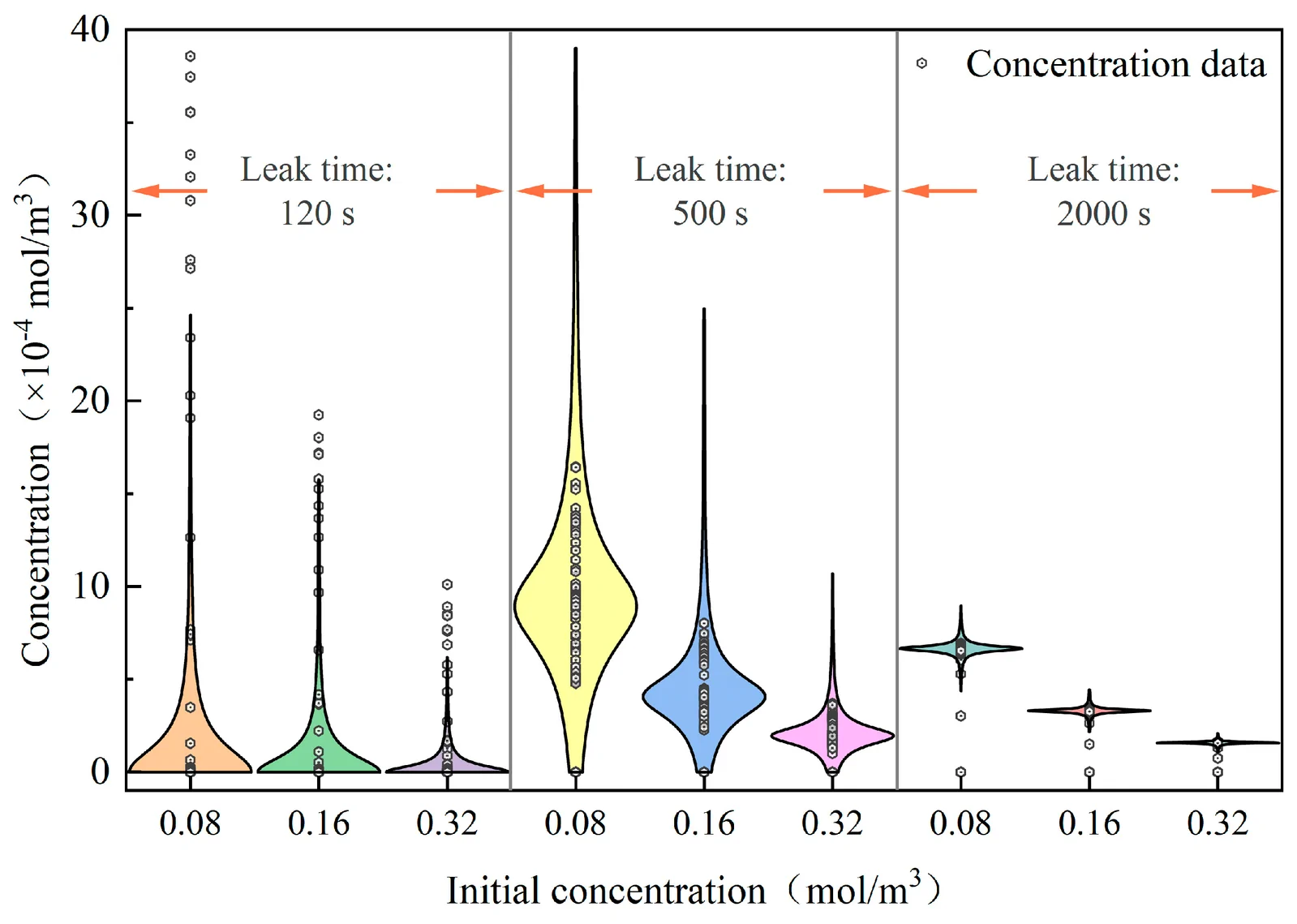
SO_2_ concentration probability distribution vs. release time under different initial concentrations.

Figure 5 shows the concentration probability distribution of SO_2_ at the main sampling port under a fault near the basin insulator for three initial concentrations: 0.08 mol/m^3^, 0.16 mol/m^3^, and 0.32 mol/m^3^. It can be seen that at 120 s, the concentrations under all conditions are mainly distributed in the low-value range, accompanied by a small number of scattered high-concentration points. This indicates that, at the initial stage of release, SO_2_ does not yet reach the sampling port consistently, and the local response still fluctuates considerably. At 500 s, the overall concentration distribution shifts upward, indicating that SO_2_ has been transported to the vicinity of the main sampling port. At 2000 s, the box range for each condition becomes significantly narrower, and the median values tend to converge, suggesting that the concentration distribution in the gas chamber gradually becomes more uniform after long-term diffusion.

Overall, the concentration distribution of SO_2_ evolves from a dispersed pattern to a more concentrated one over time. The initial concentration has a more pronounced effect on the early-stage response, while its influence gradually weakens at later stages.

**Figure 6:**
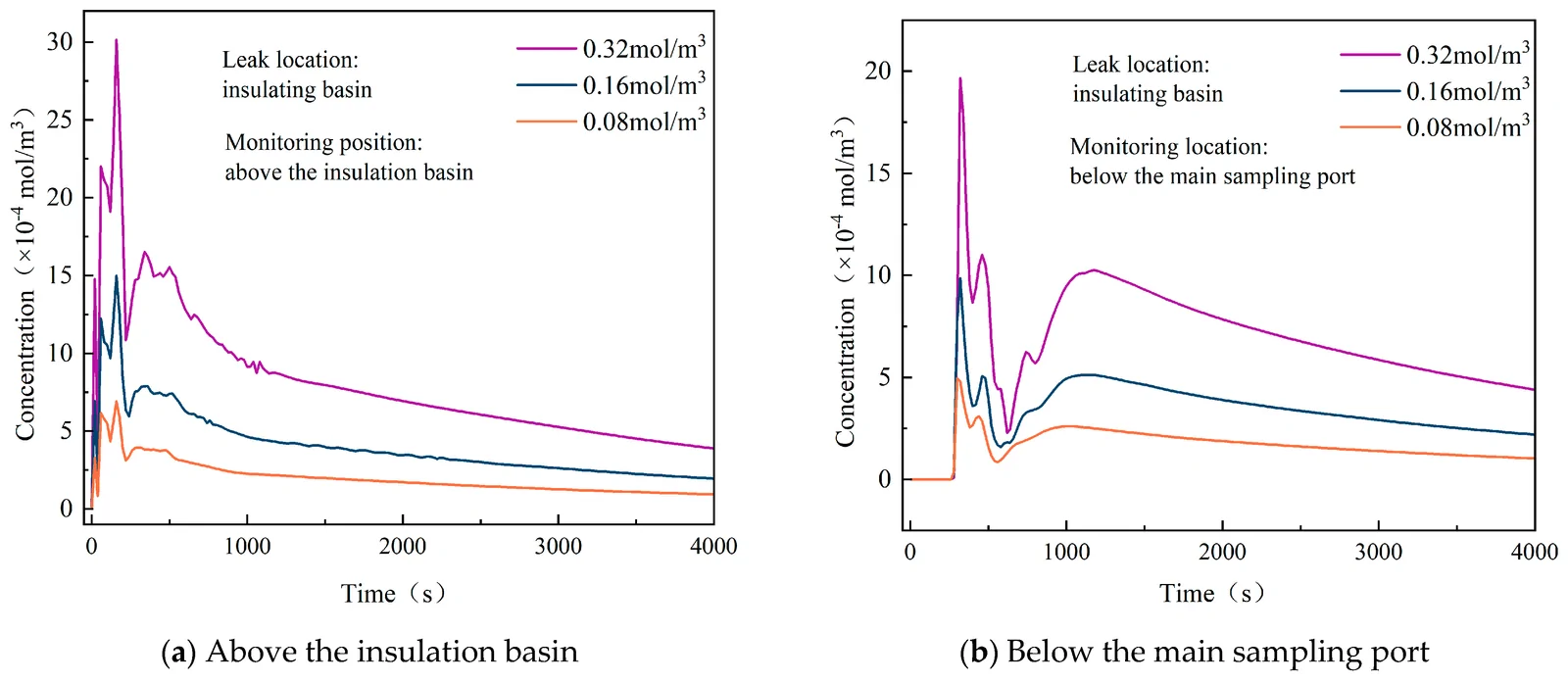
Time-dependent distribution of SO_2_ at different initial concentrations at the insulating basin. (**a**) above the insulation basin, and (**b**) below the main sampling port.

Figure 6 presents the time-varying concentration curves of SO_2_ at the locations above the basin insulator and below the main sampling port for three initial concentrations, 0.08 mol/m^3^, 0.16 mol/m^3^, and 0.32 mol/m^3^, when the fault source is located near the basin insulator. It can be seen that, at the initial stage of fault release, the concentration under all conditions rises rapidly and reaches a peak, and then gradually decreases due to diffusion and dilution. A higher initial concentration corresponds to a higher peak concentration and maintains a higher concentration level throughout the diffusion process, indicating that the initial concentration directly affects the response intensity at the monitoring points.

A comparison of Figure 6a,b shows that the peak at the monitoring point above the basin insulator appears earlier and exhibits more pronounced fluctuations, whereas the concentration response at the point below the main sampling port is relatively delayed and the curve changes more smoothly. This indicates that the monitoring point near the fault source is more sensitive to early-stage leakage, while the main sampling port, being farther from the fault source, primarily reflects the concentration variation after axial diffusion of the decomposition products.

As time progresses, the concentrations under all conditions gradually decrease and tend to stabilize. The differences caused by the initial concentration become smaller, although they do not disappear completely. Taken together, Figure 5 and Figure 6 show that the influence of the initial concentration on the SO_2_ diffusion process is mainly manifested in the early response stage, where it changes the peak concentration at the monitoring points and the degree of dispersion in the local concentration distribution. As diffusion and dilution continue, the concentration differences among the different initial concentration cases gradually diminish, and the SO_2_ distribution in the gas chamber tends to become more uniform. This indicates that the effect of initial concentration is more significant on the transient monitoring response than on the long-term steady distribution.

### 3.4. Experimental Validation of Simulation Results

#### 3.4.1. Experimental Platform Setup

**Figure 7:**
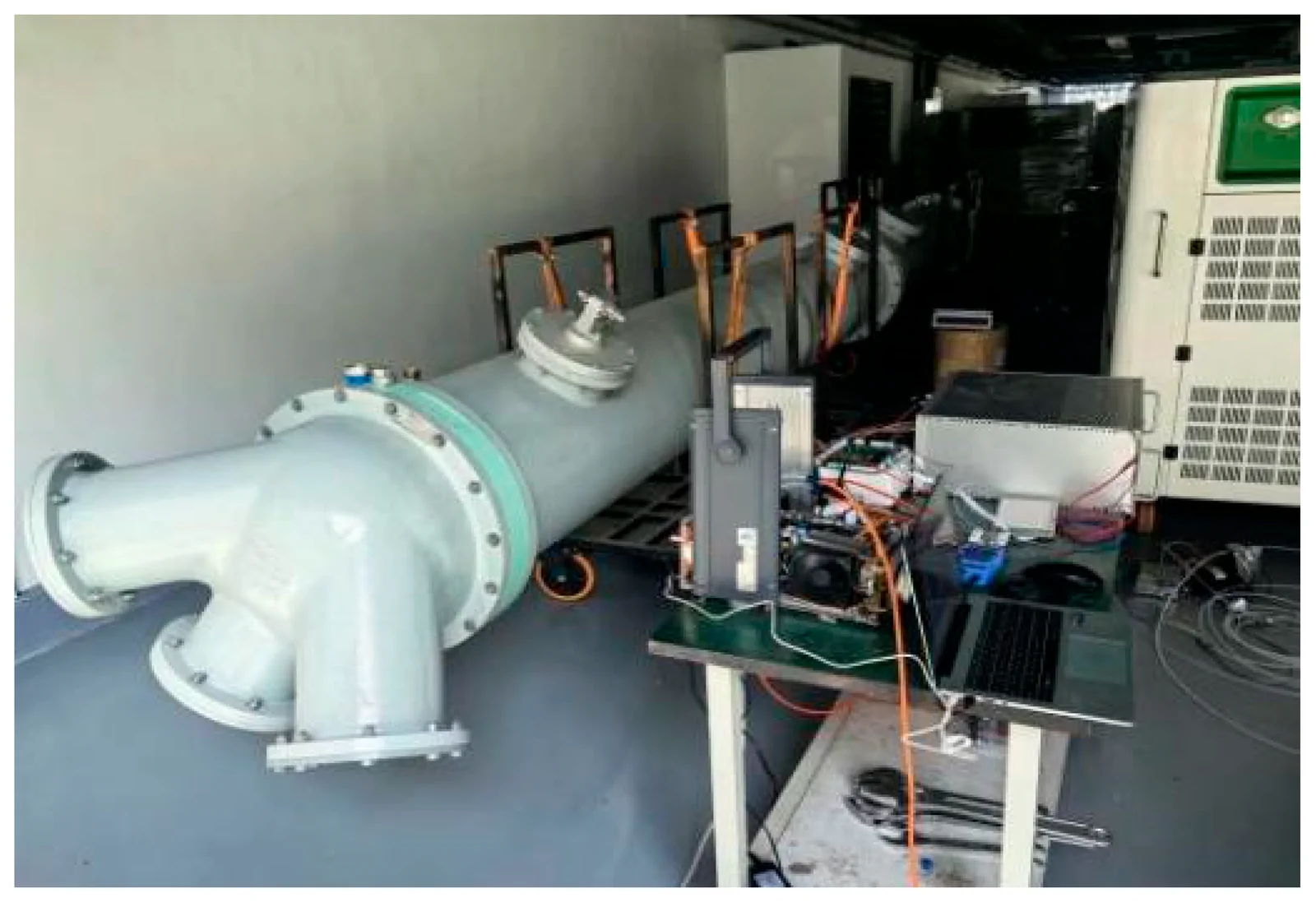
GIS gas chamber simulation test platform.

To verify the predictive reliability of the simulation model for the diffusion process of SF_6_ decomposition products in the GIS busbar gas chamber, an equivalent GIS busbar gas chamber diffusion experimental platform was established, as shown in Figure 7. The platform mainly consists of a simulated gas chamber, a temperature control system, a quantitative decomposition product release system, a multi-point concentration sampling system, and a data acquisition system.

**Figure 8:**
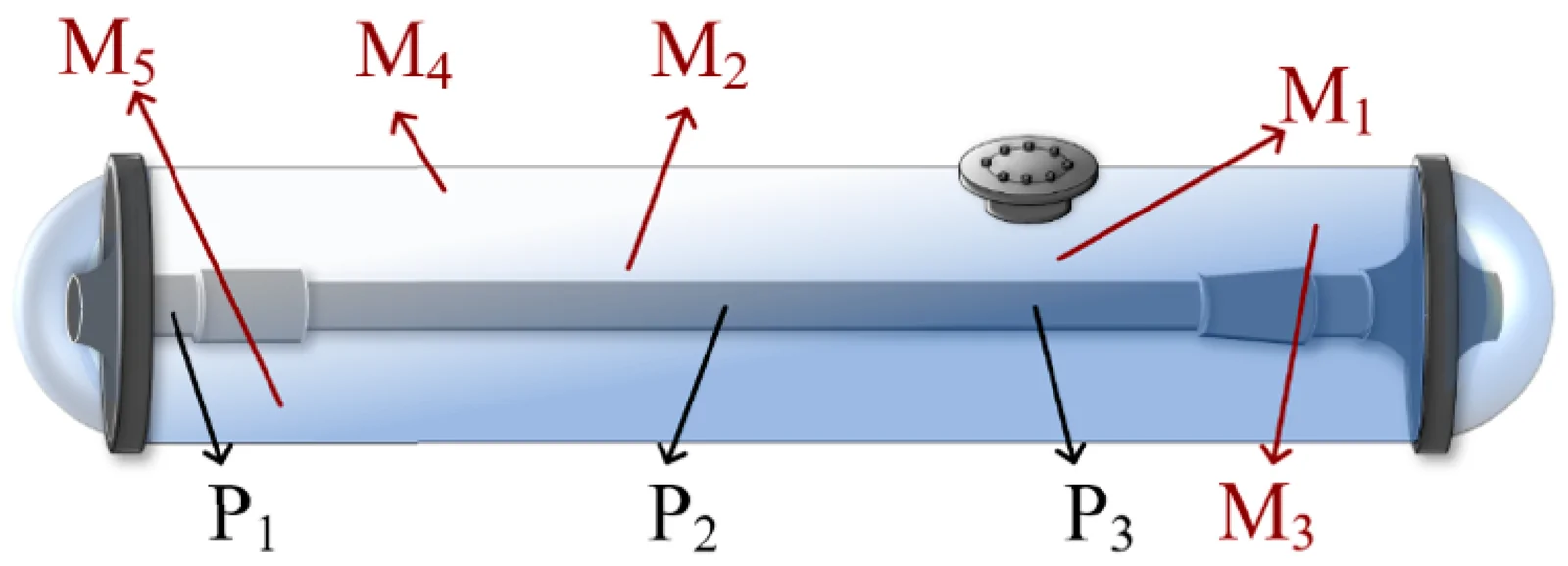
Schematic diagram of defect locations and monitoring positions.

The simulated gas chamber adopts the structural form of a three-phase common-enclosure GIS busbar. Inside the chamber, three-phase conductors, the enclosure wall, and equivalent insulating support structures are arranged. Several gas release ports are installed near the bottom, middle, top, and insulator region of the gas chamber to simulate different locations of partial discharge fault sources. The specific arrangement of monitoring points and defect locations used in the simulation is listed in Table 5 and Figure 8. In this experiment, by simulating a defect location near the main sampling port, sensing detectors were arranged at the middle point, the sampling port, and the far-end point for measurement. Multiple gas sampling points were also arranged in both the axial and radial directions of the gas chamber.

The experimental conditions were set as follows: a 50 Hz sinusoidal AC voltage of 225 kV was applied, with the GIS enclosure and flanges grounded. The simulation of a single discharge pulse was performed over a time window of 0–10 μs, with an equivalent continuous discharge duration of 24 h and a tentative apparent discharge magnitude of 100 pC/pulse, which was treated as an adjustable parameter. The gas mixture was composed of bottled SF_6_ with a purity of 99.99%, with initial H_2_O and O_2_ concentrations of 100 ppmv and 100 ppmv, respectively, and the balance being SF_6_. The absolute pressure was maintained at 0.6 MPa at 20 °C. For gas sampling, a continuous extraction at 50 mL/min over 5 min was adopted, yielding a total sampling volume of 250 mL, using a mass flow outlet without instantaneous extraction.

#### 3.4.2. Experimental Procedure

Before the experiment began, the effects of residual air and impurity gases were first eliminated by vacuum pumping or repeated gas replacement, after which SF6 background gas was filled to the specified pressure. To simulate the thermal boundary conditions in actual GIS operation, heating devices were installed inside the three-phase conductors, and isothermal or non-isothermal conditions were formed through heat dissipation from the enclosure. After the temperature field and background concentration inside the gas chamber became stable, a preset fault source location was selected, and the target gas was continuously released at a constant rate through a standard gas cylinder, a mass flow controller, and a micro-release valve.

After the experiment, the residual gas was replaced or recovered, and the release conditions, temperature data, and concentration variation results at each sampling point were compiled for subsequent comparison and validation against the simulation results.

#### 3.4.3. Experimental Results and Analysis

The root-mean-square error (RMSE) [28] was used to evaluate the agreement between the experimental and simulation results:

(6) $$RMSE_{i}=\sqrt{\frac{1}{N}\sum_{k=1}^{N}{\left[C_{i,sim}\left(t_{k}\right)-C_{i,exp}\left(t_{k}\right)\right]}^{2}}$$

**Figure 9:**
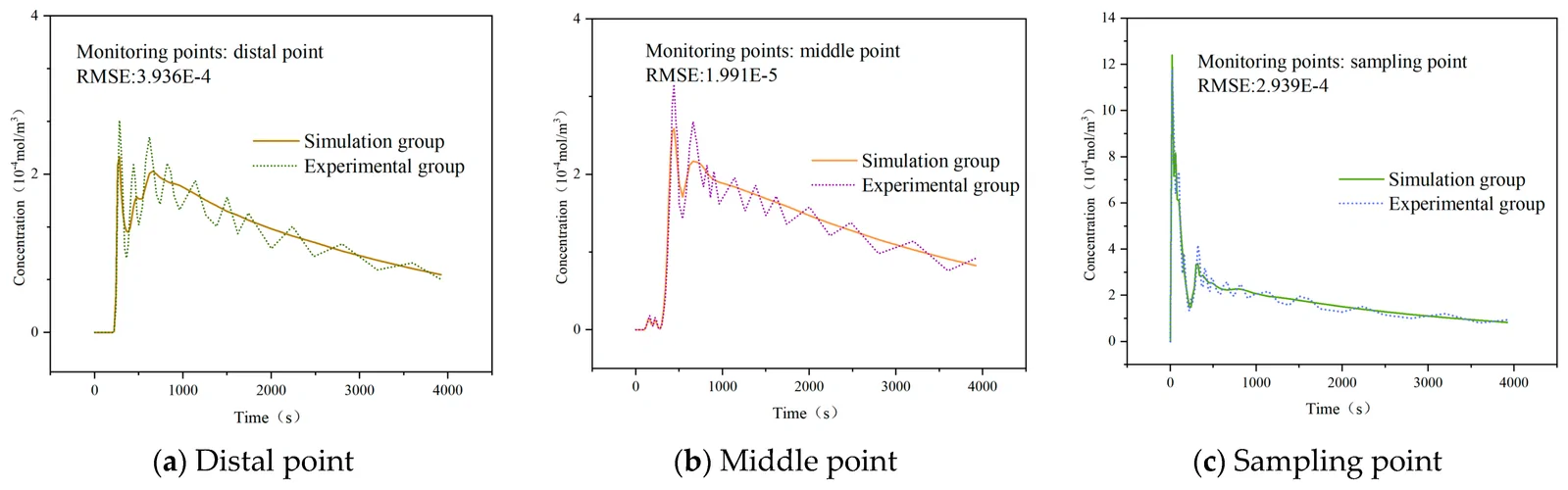
Verification of experimental and simulation results at different points (**a**) distal point, (**b**) middle point, and (**c**) sampling point.

Figure 9 presents a comparison between the experimental and simulated concentrations of SF_6_ decomposition products at the three monitoring points. It can be seen that the simulation results are in good agreement with the experimental results during the concentration rise, peak variation, and late-stage decay, indicating that the model can effectively characterize the diffusion process of decomposition products in the GIS busbar gas chamber.

The RMSE values at the distal point, middle port, and sampling point are 3.936 × 10^−4^, 1.991 × 10^−5^, and 2.939 × 10^−4^, respectively. Among them, the error at the middle point is the smallest, indicating that the model has relatively high prediction accuracy in the main region of the gas chamber. Certain deviations are observed near the peak values at the sampling port and far-end point, which may be related to local flow-field disturbances, sensor response delay, and simplification of the geometric model.

Overall, the simulation model demonstrates good prediction accuracy and reliability, and it can be used for subsequent monitoring point optimization and fault diagnosis analysis.

## 4. Comprehensive Evaluation and Optimization of Sensor Placement

### 4.1. Construction of Evaluation Indicators

To optimize the arrangement of monitoring points for decomposition products in the GIS busbar gas chamber, a comprehensive evaluation index system for sensor placement was constructed based on the response characteristics of each candidate monitoring point under different fault source locations. Considering the response speed, detection sensitivity, and fault coverage capability of the monitoring points for fault gases, the average response time, average peak concentration, and fault coverage rate were selected as the evaluation indicators.

First, the response time was used to characterize the rapid sensing capability of a monitoring point for fault gases. By simulating the concentration response processes of monitoring points M1–M5 under different fault source locations (P1, P2, and P3), the first-arrival response time of each monitoring point was extracted, and a fault-source–monitoring-point response time matrix was constructed, as shown in Table 6.

On this basis, the average response time at each monitoring point was calculated across different fault source locations, where the response time is defined as the time elapsed until the concentration exceeds the sensor detection threshold of 0.5 ppm:

(7) $$T_{j}=\frac{1}{n}\sum_{i=1}^{n}t_{ij}$$

where T_j_ is the average response time of the j-th monitoring point; t_ij_ is the response time of the j-th monitoring point under the i-th fault source; and n is the number of fault sources, with *n* = 3 in this study. The calculation results show that M2 has the shortest average response time, at 87.99 s, followed by M4, at 124.34 s. In contrast, the responses of M3 and M5 are significantly delayed, with average response times of 502.83 s and 638.28 s, respectively.

Second, the peak concentration was used to characterize the concentration response intensity of each monitoring point to decomposition products. According to the concentration–time curves of each monitoring point under different fault source locations, the peak concentrations were extracted and a peak concentration matrix was constructed, as shown in Table 7.

The average peak concentration of each monitoring point was calculated as follows:

(8) $$C_{j}=\frac{1}{n}\sum_{i=1}^{n}c_{ij}$$

where C_j_ is the average peak concentration of the j-th monitoring point, and c_ij_ is the peak concentration of the j-th monitoring point under the i-th fault source. The calculation results show that M4 has the highest average peak concentration, reaching 5.0422 mol·m^−3^, indicating that it has the capability for a strong concentration response to fault gases. In contrast, M5 has the lowest average peak concentration, at 3.8235 mol·m^−3^, suggesting relatively weak detection sensitivity.

Finally, the fault coverage rate was used to characterize the ability of a monitoring point to cover different fault source locations. In the simulations, the monitoring points arranged at different locations could all reach the response state within a certain period of time. Therefore, the fault detection coverage rate of each monitoring point was 100%.

### 4.2. Weighted Comprehensive Evaluation Method

Since the evaluation criteria differ in both dimension and nature, dimensionless normalization of the raw data is required prior to analysis. Response time is a cost-type indicator, as a shorter response time reflects quicker detection at the monitoring point. In contrast, peak concentration and fault coverage factor are benefit-type indicators, as larger values correspond to superior detection capability. Moreover, under identical sensor specifications, peak concentration directly influences the signal-to-noise ratio and detection reliability, with higher concentration levels enabling earlier exceedance of the detection threshold; therefore, it also functions as a reliability indicator.

Therefore, for the response time indicator, the following cost-type normalization formula was adopted:

(9) $$X_{T_{j}}=\frac{T_{\max}{-T}_{j}}{T_{\max}{-T}_{\min}}$$

where T_max_ and T_min_ are the maximum and minimum average response times among all monitoring points, which are 638.28 s and 87.99 s, respectively.

For the peak concentration indicator, the following benefit-type normalization formula was adopted:

(10) $$X_{C_{j}}=\frac{C_{j}{-C}_{\min}}{C_{\max}{-C}_{\min}}$$

where C_max_ and C_min_ are the maximum and minimum average peak concentrations among all monitoring points, which are 5.0422 mol·m^−3^ and 3.8235 mol·m^−3^, respectively.

For the fault coverage rate, since all monitoring points have a coverage rate of 100%, their normalized scores were all taken as 1. To avoid the influence of subjective weighting on the evaluation results, the entropy weight method was used in this study to determine the weight of each indicator.

Let the normalized indicator matrix be:

(11) $$X={\left(x_{\mathrm{ij}}\right)}_{m\times n}$$

where *m* is the number of evaluation objects, which is the five monitoring points in this study (*m* = 5), and *n* is the number of evaluation indicators, which is three in this study (*n* = 3). The proportion of the i-th evaluation object under the j-th indicator is calculated as:

(12) $$p_{ij}=\frac{x_{ij}}{\sum_{i=1}^{m}x_{ij}}$$

The information entropy of the j-th indicator is calculated as follows [25]:

(13) $$e_{j}=-k\sum_{i=1}^{m}p_{ij}lnp_{ij}$$

where

(14) $$k=\frac{1}{ln\;m}$$

The coefficient of variation for the indicator is given by:

(15) $$d_{j}{=\;1\;-\;e}_{j}$$

The final weight is calculated as:

(16) $$w_{j}=\frac{d_{j}}{\sum_{j=1}^{n}d_{j}}$$

Based on the normalized results, the weights of average response time, average peak concentration, and fault coverage rate are 63.60%, 36.40%, and 0%, respectively. This indicates that the response time varies greatly among the monitoring points and has the greatest influence on the optimal monitoring point selection results, followed by the peak concentration. Since the coverage rates of all monitoring points are the same, this indicator does not participate in the ranking differentiation in this dataset.

On this basis, the comprehensive score of each monitoring point is calculated using the linear weighted method:

(17) $$S_{j}=100(w_{T}X_{T,j}+w_{C}X_{C,j}+w_{R}X_{R,j})$$

Since w_R_ = 0 the comprehensive score can be simplified as:

(18) $$S_{j}=100(0.6360X_{T,j}+0.3640X_{C,j})$$

where S_j_ is the comprehensive evaluation score of the j-th monitoring point; w_T_, w_C_, and $w_{R}$ are the weights of response time, peak concentration, and fault coverage rate, respectively; and X_T_, X_C_, and X_R_ are the normalized scores of the corresponding indicators.

### 4.3. Comprehensive Evaluation of Sensor Placement

The comprehensive evaluation results of the five candidate monitoring points are shown in Table 8. The comprehensive scores of the monitoring points, in descending order, are M4, M2, M1, M3, and M5. Among them, M4 has the highest score, reaching 95.74; M2 ranks second, with a score of 90.01; and M5 has the lowest score.

In terms of individual indicators, M2 has the shortest average response time, indicating that it is capable of exhibiting the fastest response to fault gas and is suitable for early warning. M4 has the highest average peak concentration, while its response time is second only to that of M2, indicating that it combines good detection sensitivity with a fast response speed. In contrast, M1 shows relatively balanced performance across all indicators, but its advantages are less significant than those of M4 and M2. M3 and M5 have longer average response times, and M5 also exhibits a relatively low peak concentration response; therefore, they are not suitable as priority locations for single-sensor deployment.

The comprehensive evaluation results indicate that, given that the fault coverage factor is 100% for all candidate monitoring points, the optimization of sensor placement is primarily governed by response time and peak concentration. The response time weight (63.60%) is higher, suggesting that rapid response capability is a dominant factor in single-sensor placement optimization.

To further verify the robustness of the evaluation results, a sensitivity analysis of the weight coefficients was conducted. Under the constraint that the sum of the response time weight and the peak concentration weight equals 1, the variation in the comprehensive scores was examined as the response time weight varied from 0.5 to 0.9. The analysis reveals that M4 maintains the highest comprehensive score when the response time weight is no less than 0.55. When the response time weight falls below 0.5, M2 surpasses M4 in the comprehensive score. The response time weight determined by the entropy weight method in this study is 0.636, which falls within the robust interval in which M4 remains the top-ranked monitoring point. Furthermore, within the range of 0.55 to 0.75 for the response time weight, M4 consistently outperforms M2. This confirms that our conclusion regarding M4 as the optimal single-sensor placement is insensitive to the choice of weight coefficients and exhibits satisfactory robustness. Notably, M2 consistently maintains the second-highest ranking across different weight settings, further supporting its suitability as an auxiliary monitoring point.

## 5. Conclusions

In this paper, the diffusion process of SF_6_ decomposition products in a GIS busbar gas chamber was investigated. A numerical simulation model considering fault source location, decomposition product type, and initial concentration was established, and the reliability of the model was verified using a simulated experimental platform. On this basis, indicators including response time, peak concentration, and fault coverage rate were introduced, and the entropy weight method combined with the linear weighted evaluation method was used to comprehensively evaluate the candidate monitoring points and optimize sensor placement. The main findings are as follows:

1. The diffusion behavior of SF_6_ decomposition products in the GIS busbar gas chamber is jointly affected by fault source location, gas properties, and initial concentration. The fault source location mainly determines the concentration response speed and spatial distribution pattern. Faults near the main sampling port show the fastest monitoring response and relatively high peak concentrations; faults near the insulating basin tend to form an obvious unidirectional concentration gradient; and faults in the middle of the chamber exhibit relatively balanced diffusion with stronger symmetry in concentration distribution. Different fault locations mainly change the concentration response intensity and spatial distribution pattern, but do not significantly alter the relative concentration relationships among the decomposition products.
2. The diffusion differences among different decomposition products are jointly controlled by physical parameters such as density, dynamic viscosity, and diffusion coefficient. SO_2_ shows a strong local accumulation tendency, HF has weak migration capability and tends to remain near the fault source, H_2_S is more likely to migrate and diffuse within the gas chamber, and SOF_2_ presents a relatively uniform distribution. The initial concentration mainly affects the concentration gradient and non-uniformity in the early diffusion stage. As diffusion proceeds, the concentration distributions under different working conditions gradually become stable.
3. The simulated curves are generally consistent with the experimental curves in the concentration rise, peak variation, and later attenuation stages, indicating that the established model can accurately describe the diffusion characteristics of SF_6_ decomposition products in the GIS busbar gas chamber. The sensor placement optimization results show that the fault coverage rates of all candidate monitoring points are 100%, and response time and peak concentration are the main factors affecting the optimization results. Among them, M4 obtains the highest comprehensive score, combining fast response speed with high concentration response capability. Therefore, it is suitable as the preferred single-sensor placement point and can provide a reference for defect detection and sensor arrangement in GIS gas chambers.

## Figures and Tables

**Table 1:** Main structural parameters of busbar air chamber.

Parameter Name	Symbol	Size/mm
GIS total length	L	2930
Enclosure outer diameter	D_w_	504
Enclosure inner diameter	D_s_	480
Conductor length	L_c_	2680
Conductor diameter	D_c_	228.6
Flange diameter	D_f_	1260
Main sampling port diameter	D_v_	348

**Table 2:** Gas physical property parameters.

Material	Density/(kg·m^−3^)	Thermal Conductivity/(W·m^−1^·K^−1^)	Dynamic Viscosity/(Pa·s)	Specific Heat Capacity at Constant Pressure/(J·kg^−1^·K^−1^)
SF_6_	23.57	1.21 × 10^−2^	1.42 × 10^−5^	6.65 × 10^2^
SO_2_	10.34	9.71 × 10^−3^	1.28 × 10^−5^	6.21 × 10^2^
H_2_S	5.50	1.36 × 10^−2^	1.24 × 10^−5^	1.04 × 10^−3^
SOF_2_	13.89	1.06 × 10^−2^	1.32 × 10^−5^	6.44 × 10^−2^
HF	0.92	2.35 × 10^−2^	1.14 × 10^−2^	4.04 × 10^−4^
Conductor	2730	15,500	/	8.93 × 10^2^
Enclosure	2700	20,100	/	9.00 × 10^2^

**Table 3:** Gas diffusion coefficients.

Parameter	SF_6_	SO_2_	H_2_S	SOF_2_	HF
Diffusion coefficient/(m^2^·s^−1^)	/	4.78 × 10^−6^	6.48 × 10^−6^	4.04 × 10^−6^	1.35 × 10^−5^

**Table 4:** Comparison of key parameters under different mesh schemes.

Defect Location	Number of Mesh Elements/Ten Thousand	*T*_max_/K	*T*_max_ Relative Variation/%	*u*_max_/(m·s^−1^)	*u*_max_ Relative Variation/%
Main sampling port	23	769.23	/	0.224	/
	48	771.11	0.24	0.281	25.446
	83	772.12	0.13	0.312	11.032
	120	772.25	0.02	0.313	0.321
Middle of the chamber	23	767.95	0.56	0.231	26.198
	48	770.24	0.30	0.279	20.779
	83	772.99	0.36	0.314	12.545
	120	772.96	0.01	0.315	0.318
Insulating basin	23	768.45	0.59	0.229	27.302
	48	770.67	0.29	0.285	24.454
	83	772.45	0.23	0.309	8.421
	120	772.63	0.02	0.311	0.647

**Table 5:** Positions of defects and monitoring points.

ID	Type	Location Description
P1	Defect location	Near the main sampling port
P2	Defect location	Middle of the chamber
P3	Defect location	Deep area/behind the insulator
M1	Monitoring point	Near the main sampling port
M2	Monitoring point	Middle main channel
M3	Monitoring point	Distant connecting chamber
M4	Monitoring point	Upper space
M5	Monitoring point	Local side-wall stagnant zone

**Table 6:** Response time matrix for different fault sources and monitoring points.

Fault Source	M1/s	M2/s	M3/s	M4/s	M5/s
P1	32.58	88.65	312.66	140.40	562.83
P2	112.48	89.10	461.96	102.52	578.88
P3	420.18	74.21	734.86	132.09	774.13

**Table 7:** Peak concentration matrix for different fault sources and monitoring points.

Fault Source	M1/mol·m^−3^	M2/mol·m^−3^	M3/mol·m^−3^	M4/mol·m^−3^	M5/mol·m^−3^
P1	4.9852 × 10^−4^	5.9267 × 10^−4^	6.8330 × 10^−4^	6.7742 × 10^−4^	5.6844 × 10^−4^
P2	3.2065 × 10^−4^	2.3245 × 10^−4^	3.4638 × 10^−4^	2.6690 × 10^−4^	3.0939 × 10^−4^
P3	5.9675 × 10^−4^	5.8717 × 10^−4^	3.2705 × 10^−4^	5.6833 × 10^−4^	2.6921 × 10^−4^

**Table 8:** Comprehensive score for single-sensor placement.

Monitoring Point	Response Time Score	Peak Concentration Score	Coverage Rate Score	Comprehensive Score
Weight	0.6360	0.3640	/	/
M1	0.8130	0.7354	1.0000	78.48
M2	1.0000	0.7255	1.0000	90.01
M3	0.2461	0.5735	1.0000	36.53
M4	0.9330	1.0000	1.0000	95.74
M5	0.0000	0.0000	1.0000	0.00

## Data Availability

Data are not available for privacy reasons.
